# Aquaporin Membrane Channels in Oxidative Stress, Cell Signaling, and Aging: Recent Advances and Research Trends

**DOI:** 10.1155/2018/1501847

**Published:** 2018-03-27

**Authors:** Grazia Tamma, Giovanna Valenti, Elena Grossini, Sandra Donnini, Angela Marino, Raul A. Marinelli, Giuseppe Calamita

**Affiliations:** ^1^Department of Biosciences, Biotechnologies and Biopharmaceutics, University of Bari Aldo Moro, Bari, Italy; ^2^Department of Translational Medicine, University of Eastern Piedmont, Novara, Italy; ^3^Department of Life Sciences, University of Siena, Siena, Italy; ^4^Department of Chemical, Biological, Pharmaceutical and Environmental Sciences, University of Messina, Messina, Italy; ^5^Instituto de Fisiología Experimental, CONICET, Facultad de Ciencias Bioquímicas y Farmacéuticas, Universidad Nacional de Rosario, Rosario, Santa Fe, Argentina

## Abstract

Reactive oxygen species (ROS) are produced as a result of aerobic metabolism and as by-products through numerous physiological and biochemical processes. While ROS-dependent modifications are fundamental in transducing intracellular signals controlling pleiotropic functions, imbalanced ROS can cause oxidative damage, eventually leading to many chronic diseases. Moreover, increased ROS and reduced nitric oxide (NO) bioavailability are main key factors in dysfunctions underlying aging, frailty, hypertension, and atherosclerosis. Extensive investigation aims to elucidate the beneficial effects of ROS and NO, providing novel insights into the current medical treatment of oxidative stress-related diseases of high epidemiological impact. This review focuses on emerging topics encompassing the functional involvement of aquaporin channel proteins (AQPs) and membrane transport systems, also allowing permeation of NO and hydrogen peroxide, a major ROS, in oxidative stress physiology and pathophysiology. The most recent advances regarding the modulation exerted by food phytocompounds with antioxidant action on AQPs are also reviewed.

## 1. Introduction

Reactive oxygen species (ROS) are unstable reactive molecules, physiologically produced by xanthine oxidase, nicotinamide adenine dinucleotide phosphate oxidase, lipoxygenases, and mitochondria [1, 2]. Though oxygen is peremptory for life, imbalances between antioxidant defense mechanisms, overproduction of ROS, or incorporation of free radicals from the environment to living systems lead to oxidative stress. ROS and other reactive species are implicated in a large spectrum of biological conditions, such as mutation, tumorigenesis, degenerative diseases, inflammation, aging, frailty, and development [3]. ROS exert a dual role as both deleterious and beneficial species, the latter being of pivotal importance as signaling molecules. At physiological levels, ROS can improve cellular activities as they are involved in the control of the chemical balance and synaptic plasticity [4], whereas an excess amount of ROS can damage the endothelium, leading to alteration of the intracellular reduction-oxidation homeostasis [5].

Among various mechanisms, the uncoupling of nitric oxide synthase (NOS) in vascular cells has also widely been reported to be involved in ROS generation. In that event, NOS is turned into a peroxynitrite generator, leading to detrimental effects on vascular function, due to lipidic peroxidation [6]. Furthermore, superoxide anions can modify endothelial function by reducing nitric oxide (NO) biosynthesis and bioavailability [7]. This issue is of particular relevance since changes in NO release could play an important role in endothelial function maintenance, in addition to regulating proliferation of smooth muscle cells, leukocyte adhesion, platelet aggregation, angiogenesis, thrombosis, vascular tone, and hemodynamics. Hence, endothelial dysfunction, a predictor of several cardiovascular diseases (CVDs), is caused by imbalance between vasodilating and vasoconstricting agents, including NO, endothelium-derived hyperpolarizing factor, prostacyclin, or vasoconstrictive factors such as thromboxane (TXA_2_) and endothelin-1 (ET-1) [8].

NO is a gas which plays an important role in blood pressure modulation due to its signaling action on renal, cardiovascular, and central nervous system functions [9]. The role of NO in vascular homeostasis also comes from the negative regulation on coagulation and inflammation operated by this signaling molecule.

Throughout the years, ROS and NO have been widely considered to enter cells by freely diffusing through the cell membrane lipid bilayer and not via specific transporters or channels. This notion has been challenged by the discovery of new membrane transport functions, especially those exerted by aquaporins (AQPs), a family of membrane channel proteins widespread in nature [10, 11]. Transport of NO and ROS by AQPs would be required for cell homeostasis to play a critical role in maintaining endothelial function.

This review focuses on an emerging topic, the functional involvement of AQPs in ROS membrane transport, with specific regard to the movement of hydrogen peroxide and NO into and out of cells, in both health and oxidative stress-induced diseases. The emerging information and research trends regarding the modulation exerted by food phytocompounds with antioxidant action on the expression and function of AQPs are also reviewed.

## 2. Exogenous and Endogenous Source of Oxidants

Reactive species (RS) derive from either endogenous or exogenous sources. Prolonged exercise, ischemia, inflammation, infection, cancer, and aging correlate with production of free radicals. Production of ROS and reactive nitrogen species (RNS) may occur through enzymatic and nonenzymatic reactions [12, 13]. Among enzymatic processes, NADPH oxidase (NOX), xanthine oxidase, and peroxidases play a pivotal role in free radical generation. For example, NOX catalyzes the production of superoxide [14], which represents a master substrate for generation of other RS, such as hydrogen peroxide (H_2_O_2_), hydroxyl radical (OH^•^), peroxynitrite (ONOO^−^), and hypochlorous acid (HOCl). The latter is synthesized in neutrophils by myeloperoxidase, an enzyme oxidizing chloride ions when H_2_O_2_ is present [15, 16]. Nitric oxide (NO^•^) is generated in many tissues and results from the oxidation of l-arginine to citrulline through the action of nitric oxide synthase [17], as reported above.

Nonenzymatic reactions can also occur during oxidative phosphorylation in mitochondria, the main RS production site inside the cell [18]. The leakage of electrons at complex I, complex II, or complex III associates with superoxide production. In the mitochondrial matrix and in the cytosol, superoxide can be converted into H_2_O_2_ by superoxide dismutase and further detoxified by catalases. In addition, ROS stimulates the generation and the release of other RS, thereby causing a vicious circle due to increased permeability of mitochondrial pores by ROS resulting in mitochondrial defects leading to release of further RS [19].

Alternatively, RS also result from reaction with organic compounds subjected to ionizing radiations. Indeed, high doses of ionizing radiation increase the production and release of inflammatory chemokines and RS that, in concert, promote tissue injury [20].

Exogenous RS can originate from water and air pollution, cigarette smoke, pesticides, dioxin, and several drugs. Once in the body, these different compounds are metabolized, generally in the liver, generating free radicals.

## 3. Aquaporins, Membrane Channel Proteins of Pleiotropic Relevance

Aquaporins (AQPs) are channel proteins widely present in living organisms where they were initially reported to facilitate the transport of water and certain neutral solutes across biological membranes [21, 22]. Mammals possess thirteen distinct AQPs (AQP0–12) that are roughly subdivided into *orthodox aquaporins* (AQP0, AQP1, AQP2, AQP4, AQP5, AQP6, and AQP8) and *aquaglyceroporins* (AQP3, AQP7, AQP9, and AQP10). Orthodox AQPs were initially described to conduct only water, whereas aquaglyceroporins were shown to transport water and some small neutral solutes, particularly glycerol. These peculiarities did not apply to AQP11 or AQP12, due to their distinct evolutionary pathway and primary sequence distinctions, the reason why they have been indicated as *unorthodox aquaporins* [23]. The transport properties and subcellular localization of AQP11 and AQP12 remain unclear and a matter of debate. The functional subdivision of AQPs has become more articulated in the light of transport properties reported in recent years. Some AQPs are also able to conduct H_2_O_2_ and/or ammonia [24], and, due to these biophysical properties, they are also denoted as *peroxiporins* [25, 26] and *ammoniaporins* (or *aquaammoniaporins*) [25, 27, 28], respectively. The currently identified mammalian AQP homologues allowing passive diffusion of considerable amounts of H_2_O_2_ are AQP1, AQP3, AQP5, AQP8, and AQP9 [29]. AQPs also facilitate permeation of gases such as CO_2_, NO, or O_2_ [11, 30, 31], features that have raised a lot of interest due to the potential physiological relevance they may have in permeating gases of biological relevance. This feature would add more knowledge to the physiological importance of gas channels in nature [32].

Expression and modulation of AQPs in all body districts are the subject of intense investigation around the world. Important roles have already been ascribed to this family of membrane channels, in both health and disease [21, 22, 33] (Table 1).

## 4. Involvement of Aquaporins in the Transport System of Reactive Species

### 4.1. Aquaporin-8 as Peroxiporin Mediating Mitochondrial H_2_O_2_ Release in Hepatocytes

H_2_O_2_ is a major ROS constantly generated in mitochondria by the aerobic metabolism. Respiratory chain-linked H_2_O_2_ is produced by enzymatic dismutation of superoxide radicals [34]. Complex I generates superoxide within the mitochondrial matrix, whereas complex III generates superoxide in the intermembrane space [34, 35]. Hepatic mitochondria are not only important sources for ROS but also important key targets for their potential damage. Under physiological conditions, H_2_O_2_ is the only ROS that can move out of the mitochondria into the cytoplasm and function as a second messenger in signal transduction pathways [35, 36]. Under oxidative stress, high ROS (H_2_O_2_) levels can induce loss of mitochondrial membrane potential and mitochondrial dysfunction with the resulting triggering of cell death mechanisms [37, 38].

H_2_O_2_ had been long thought to be freely diffusible across cellular membranes, a notion that has been challenged by both the existence of H_2_O_2_ gradients across biological membranes [39, 40] and the finding that membrane permeability is a rate-limiting factor in H_2_O_2_ elimination by mammalian cells [41]. Limited diffusion of H_2_O_2_ across mitochondrial membranes has also been suggested [42]. Hence, a protein-facilitated diffusional pathway for H_2_O_2_ across membranes was proposed [40, 42]. H_2_O_2_ size and chemical and physicochemical properties are similar to those of water [40], which may explain H_2_O_2_ passage through channel membrane proteins such as AQPs. Accordingly, initial studies in reconstituted yeast [10] and transfected mammalian cells [43] indicate that AQP8 and some other members of the mammalian AQP family facilitate H_2_O_2_ passage across plasma membranes. Thus, AQP8 is able to function as peroxiporin.

An initial study demonstrated that AQP3 is required for (NOX)-derived H_2_O_2_ signaling [43]. More recent studies in diverse nonhepatic cells have reported that plasma membrane AQP8 transports NOX-generated H_2_O_2_ that participates in intracellular signal transduction pathways [44–47]. In HeLa cells, AQP8 plays a key role in the epidermal growth factor- (EGF-) induced entry of H_2_O_2_, which in turn initiates intracellular signaling by tyrosine phosphorylation of target proteins [44]. In B lymphocytes, AQP8-mediated H_2_O_2_ transport has been reported to induce cell activation and differentiation [45], whereas in leukemia cells, it has been found to induce proliferation pathways [46, 47].

In hepatocytes, plasma membrane AQP8 is exclusively expressed on the bile canalicular domain [48]. Therefore, AQP8 cannot be involved in the intracellular transport of H_2_O_2_ generated by NADPH oxidases at sinusoidal plasma membranes. AQP8 is also expressed in the inner mitochondrial membranes of some cells, including hepatocytes [49, 50]. Experimental evidence in human hepatocyte carcinoma HepG2 cells suggests that mitochondrial AQP8 (mtAQP8) facilitates the diffusional efflux of H_2_O_2_ [51]. A similar observation was made studying mitochondrial AQP8b, the marine teleost orthologue of human AQP8 [52]. As reviewed below, the involvement of an mtAQP8-mediated H_2_O_2_ transport in normal human spermatozoa functioning has also been suggested [53].

The knockdown of mtAQP8 expression in HepG2 cells markedly reduces the release of mitochondrially generated H_2_O_2_, and the resulting mitochondrial ROS accumulation induces mitochondrial depolarization via the mitochondrial permeability transition mechanism and reduced ATP levels [51]. Interestingly, the immunological blockage of AQP8b-mediated mitochondrial H_2_O_2_ efflux in marine spermatozoa also causes ROS accumulation, mitochondrial depolarization, and decreased ATP production [52].

The oxidant-induced mitochondrial dysfunction in HepG2 cells causes loss of viability by activating a necrotic death pathway [51, 54]. Interestingly, mtAQP8 silencing causes a minor loss of viability in human hepatoma HuH-7 cells but does not affect viability in neither in normal rat hepatocytes nor in the nonneoplastic human cell lines, renal HK-2, and Chang liver cells [54]. Therefore, carcinoma cells might be particularly susceptible to defective mtAQP8 expression. As the loss of viability in mtAQP8-knockdown HepG2 cells is prevented by the mitochondria-targeted antioxidant MitoTempol [51], a disparity in mitochondrial antioxidant defenses is likely to explain the observed differential susceptibility among mtAQP8-knockdown cells. Nevertheless, it is worth mentioning that, at least for total and reduced mitochondrial glutathione levels, there were no significant differences between HepG2, HuH-7, Chang liver cells, and rat hepatocytes (unpublished data from Raul A. Marinelli's laboratory). Further studies are needed to understand the mechanisms that actually cause differential death in mtAQP8-knockdown cells.

With the use of HeLa cells, the AQP8-mediated plasma membrane H_2_O_2_ transport has recently been reported to be functionally modulated under stress [55]. AQP8 permeability to H_2_O_2_ was reversibly inhibited, thus preventing intracellular ROS accumulation during oxidative stress [55]. To the best of our knowledge, as AQP8 expression has not been demonstrated in HeLa cell mitochondria [44, 56], it would be interesting to explore whether hepatocyte mtAQP8 is under this novel regulatory mechanism of cell survival during stress.

Another as-yet-unexplored area of research is the role that mtAQP8-mediated H_2_O_2_ may play in hepatocyte physiology. We have recently provided evidence suggesting that hepatocyte mtAQP8 expression can be modulated by cholesterol via sterol regulatory element-binding protein (SREBP) transcription factors; that is, mtAQP8 is upregulated in cholesterol-depleted cells and downregulated in cholesterol-loaded cells [57]. As H_2_O_2_ has been described to stimulate hepatocyte cholesterogenesis via SREBPs [58], our finding might suggest that mtAQP8 plays a role in SREBP-controlled cholesterol biosynthesis. For example, at low cellular cholesterol levels, SREBP-dependent mtAQP8 upregulation could facilitate the mitochondrial H_2_O_2_ release that would contribute to stimulating cholesterogenesis. Further studies are required to elucidate this issue.

### 4.2. AQP-Mediated H_2_O_2_ Transport Is Critical in Sperm Cell Motility and ROS Scavenging

The relevance of AQP-mediated water and H_2_O_2_ transport in human sperm cells activity has been reported in a recent study investigating the expression, distribution, and role of AQP3, 7, 8, and 11 in subfertile compared with normospermic subjects [53]. The investigated AQPs were found to be implicated in sperm cell volume regulation and ROS scavenging, two functions of critical importance in sperm counts and motility. With the use of AQP blockers, it was suggested that chronic deficiency in AQP-mediated H_2_O_2_ permeability impairs ROS efflux out of sperm cells and reduces the detoxification efficiency, with consequent loss of sperm functionality. However, although coordinated action of AQPs has been reported to regulate sperm motility in the marine teleost seabream [59], further studies are needed to confirm the suggested pathophysiological relevance of AQPs in human male fertility. The specific AQP homologue that, among AQP3, 7, 8, and 11, may account for sperm cell permeability to H_2_O_2_ remains elusive. AQP8 features one of the highest conductances to H_2_O_2_ among peroxiporins. However, the relevance of AQP8 as the major H_2_O_2_ membrane transport system in human sperm cells remains to be proved. A recent study using HeLa cells showed reduction of AQP-mediated water and H_2_O_2_ cell permeability following oxidative stress [60]. Interestingly, the diminution was prevented or reversed when the cells were treated with antioxidant phytochemical compounds.

### 4.3. AQP3 Mediates Hydrogen Peroxide-Dependent Intracellular Signaling, Responses to Environmental Stress, and Cell Migration

AQP3 is also reported to facilitate the uptake of H_2_O_2_ into mammalian cells [43]. Microimaging studies using peroxy yellow 1 methyl-ester (PY1-ME), a specific fluorescent probe for H_2_O_2_, showed AQP3-mediated uptake of H_2_O_2_ in HEK cells [43]. Moreover, it has been demonstrated that T-cell migration towards chemokines is regulated by AQP3-facilitated transport of H_2_O_2_ that, in turn, stimulates Rho signaling [61]. In primary keratinocytes, H_2_O_2_ is required to stimulate NF-*κ*B signaling in response to TNF-alpha [62].

Conversely, oxidative signals seem to be important in controlling AQP3 expression. Chrysin and resveratrol, two antioxidant phytocompounds, have been reported to modulate the expression of AQP3 [63, 64]. Accordingly, severe ultraviolet A (UVA) irradiation causes a significant reduction in AQP3 expression secondary to increased oxidative stress [65]. In this regard, a negative correlation between AQP3 expression and age in sun exposed skin has been described, suggesting AQP3 as a biomarker of age-related skin alteration [66].

In the colon, AQP3 is expressed in the epithelial cells where changes in expression were found in response to inflammation, and AQP3-depleted mice experienced impaired recovery after chemical-induced colitis [67]. Interestingly, mice lacking AQP3 showed impaired healing of superficial wounds in the colon. This finding elucidates the signaling mechanism of extracellular H_2_O_2_ in colonic epithelium and suggests the implication of AQP3-mediated H_2_O_2_ transport in innate immune responses at mucosal surfaces [68]. AQP3-mediated H_2_O_2_ transport has also been described to control EGF signaling in epithelial cells [69], playing an important role in T-cell and breast cancer cell migration [70, 71]. However, the exact contribution of the AQP3-mediated H_2_O_2_ transport to these changes in cellular function remains to be fully elucidated. Involvement of AQP3 in trefoil peptide and EGF-mediated migration, a vital process in inflammatory bowel disease repair in case of excess free radical production, has also been recently shown [72].

### 4.4. AQP1-Mediated Diffusion of NO in Vasorelaxation

#### 4.4.1. Endothelial NO Release and Oxidative Stress during Aging

With aging, endothelial cells (ECs) undergo considerable remodeling processes [73, 74]. Increased endothelial permeability, alterations in the cytoskeleton, the appearance of *β*-galactosidase staining, and the expression of several cell cycle inhibitors [75] are also observed. Aging of ECs is associated with an increased release of vasoconstrictors, such as angiotensin II and endothelin, and a reduced release of vasodilators, such as NO and prostacyclin [76].

**Figure 1:**
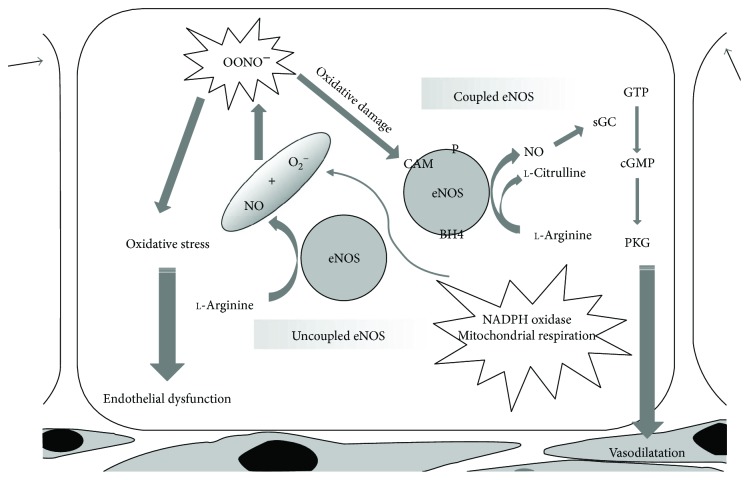
NO release by eNOS in physiological and peroxidative conditions. While “coupled” eNOS is involved in the physiological NO release underlying vasorelaxation, NO release by “uncoupled” eNOS is turned into OONO^−^ (peroxynitrites) leading to an increase in oxidative stress with consequent endothelial dysfunction. ADMA: asymmetric dimethylarginine; Akt: protein kinase B; BH4: tetrahydrobiopterin; CAM: calmodulin; Cav-1: caveolin 1; eNOS: endothelial NO synthase; cGMP: cyclic guanosine monophosphate; GTP: guanosine triphosphate; GTPCH: guanosine triphosphate cyclohydrolase I; Hsp 90: heat shock protein 90; NADPH: nicotinamide adenine dinucleotide phosphate; NO: nitric oxide; sGC: soluble guanylate cyclase; PKG: protein kinase G.

Among the above factors, NO bioavailability has been suggested to play a central role in maintaining endothelial function [77–79]. NO is the subject of extensive studies as one of the most relevant factors released by the endothelium, playing an outstanding role in maintaining vascular system function [77, 79–81]. NO is produced by endothelial NO synthase (eNOS), which transfers electrons from nicotinamide adenine dinucleotide phosphate (NADPH) to the heme in the amino-terminal oxygenase domain. In this way, the substrate l-arginine is oxidized to l-citrulline and NO. Tetrahydrobiopterin (BH4) is an essential cofactor of eNOS exerting a key role in the progression of NO synthesis (Figure 1). NO formed by the vascular ECs diffuses to the adjacent cells, such as vascular smooth muscle cells (VSMCs), platelets, and leucocytes, where it exerts many of its beneficial actions, such as vasodilation, antithrombotic, anti-inflammatory, and antiproliferative effects [82]. Endothelium-derived NO is known to be particularly important to maintain normal vascular tone, endothelial function, and homeostasis [83], preventing the progression of age-related vascular disorders [80]. Decreased production of endothelium-derived NO during aging is commonly believed to be due to decreased eNOS activity characterizing senescent ECs [78]. In the peroxidative conditions associated with aging, superoxide anion (O_2_^−^) can also react with NO leading to the formation of peroxynitrites, which, in turn, can promote protein nitration and contribute to EC dysfunction and death [84, 85]. Furthermore, enhanced oxidative stress can lead to eNOS “uncoupling” and cause endothelial dysfunction [86].

BH4 oxidation is one of the possible mechanisms of eNOS “uncoupling.” Intracellular BH4 levels depend on the balance between its synthesis and degradation. In particular, oxidative stress may lead to excessive oxidation and depletion of BH4. As a consequence, the flow of electrons within NOS could be “uncoupled” from l-arginine oxidation and O_2_^−^ produced from the oxygenase domain [87]. Hence, eNOS would be converted to a superoxide-producing enzyme with reduced NO production and enhanced preexisting oxidative stress [88, 89].

#### 4.4.2. AQP1 and NO Flow in Vascular Senescence and Atherosclerosis

Free diffusion (simple diffusion) through the phospholipid bilayer composing the plasma membrane had historically been assumed to be the only pathway whereby NO moves into or out of cells. Thus, based on the partition coefficient of NO between lipids and water [90, 91] rather than direct experimental assessment of NO diffusion across the cell membrane, NO was believed to cause vasodilation, antithrombotic, anti-inflammatory, and antiproliferative effects without need of facilitation by channels or transporters. This assumption was not confirmed after measurements of NO fluxes across reconstituted proteoliposomes and transfected cultured cells showing that, in addition to water, the AQP1 channel could conduct NO across plasma membranes and that the plasma membrane represents a significant barrier to NO diffusion [11]. Successively, with the use of thoracic aortas isolated from wild-type (*Aqp*^+/+^) and *Aqp*^−/−^ knockout mice, it was shown that AQP1 facilitates NO diffusion out of endothelial cells and NO influx into vascular smooth muscle cells, and that AQP1 conduction of NO is required for full expression of endothelium-dependent vasorelaxation [92]. Regarding vascular aging, changes in AQPs expression have been found in animal models of kidney-clip hypertension [11]. The trafficking of AQPs within cells has also been shown to change during aging, as observed in the parotid gland [93]. The suggested role of AQPs in vascular function regulation and senescence through modulation of NO diffusion across cell membranes opens a new avenue in understanding vascular senescence physiology and pathophysiology. Additional work is, however, needed since a discrepancy has been raised by a study reporting intact NO-dependent vasorelaxation in AQP1-depleted mice [94]. Vascular AQP1 expression was found to undergo positive regulation with the mediation of KLF2, the flow-responsive transcription factor Krüppel-like factor 2 that maintains an anticoagulant, anti-inflammatory endothelium with sufficient NO bioavailability [95]. Both *in vitro* and *in vivo* AQP1 expression was subjected to KLF2-mediated positive regulation by atheroprotective shear stress whereas it proved to be downregulated under inflammatory conditions. While suggesting that endothelial expression of AQP1 characterizes the atheroprotected, noninflamed vessel wall, this finding supports the putative continuous role of KLF2 in stabilizing the vessel wall via cotemporal expression of eNOS and AQP1, helping to prevent or counteract the pathogenesis of atherosclerosis.

## 5. Role of Vasopressin/AQP2 Axis and Oxidative Stress in Aging

Alterations in plasma osmolality and fluid body volume are observed in the elderly, making old people at high risk of developing disturbances of the water metabolism, which can give rise to several adverse effects. Aging blunts thirst and drinking responses, making older people more vulnerable to body fluid imbalance and dehydration [96], which can compromise cognitive function [97, 98]. Indeed, dehydration is a predisposing factor for confusion in long-term care residents [99]. Furthermore, plasma hypertonicity, a marker of dehydration, increases the risk of ischemic stroke in hospitalized patients [100] and may precipitate cerebral ischemic events in susceptible elderly individuals [101].

The major hormone regulating water metabolism in the body is vasopressin. Vasopressin is a 9-amino acid peptide that is secreted from the posterior pituitary in response to high plasma osmolality and hypovolemia. Vasopressin has important roles in circulatory and water homeostasis mediated by vasopressin receptor subtypes V1a (vascular), V1b (pituitary), and V2 (vascular, renal). Therefore, age-related dysfunction of the hypothalamic-neurohypophyseal-vasopressin axis can result in multiple abnormalities in several physiological systems that might promote a variety of morbidity such as cardiovascular and renal diseases [102, 103].

At the renal level, it has been observed that aging is accompanied by a parallel decrease in maximal urine concentrating ability [104]. Individuals aged 60–79 years show an approximately 20% reduction in maximum urine osmolality, a 50% decrease in the ability to conserve solute, and a 100% increase in minimal urine flow rate, when compared to younger age groups. Abnormalities in vasopressin secretion appear to be associated with the decrease in urine concentrating ability with aging: the abundance of many of the key transport proteins responsible for urine concentrating ability is reduced in the kidney medulla of aged rats [105]. The reductions in water, sodium, and urea transport protein abundances, along with their reduced response to water restriction, contribute to the reduced ability of aged rats to concentrate urine and conserve water [104].

The major mechanism by which vasopressin modulates water reabsorption is by regulating the trafficking of the vasopressin-sensitive water channel aquaporin-2 (AQP2) in collecting duct principal cells. Specifically, binding of vasopressin to the V2R increases cAMP levels, resulting in the activation of protein kinase A (PKA). PKA-dependent phosphorylation of the water channel AQP2, at S256, is essential to promote the translocation of AQP2-bearing vesicles from an intracellular pool to the apical plasma membrane [106]. Phosphoproteomic studies have demonstrated that, besides S256, vasopressin stimulation increases S264 and T269 but decreases the phosphorylation of S261 [107].

Several studies performed in animal models have shown that in aged rats, there is a large decrease in the level of AQP2 as well as of its phosphorylated form at S256, which can contribute to the reduced renal concentrating abilities [105, 108]. Furthermore, AQP3 is reduced in aged rats, but no change in the expression of AQP1 and AQP4 has been detected in aged rats. This distinction in the regulation of AQPs abundance may be related to the fact that only AQP2 and AQP3 expressions are under control of vasopressin.

One possible therapy to overcome the decrease in AQP2 abundance in aging might be the administration of vasopressin. A recent study showed that desmopressin (dDAVP), a selective V2R agonist, administered to 10- and 30-month-old Wag/Rij rats, decreases urine output in both rat groups and leads to an increase in AQP2 and AQP3 abundance. These results suggest that a decrease in AQP2 and AQP3 expression levels partially accounts for the diminution in urinary concentrating ability in aging.

In general, due to an impaired ability to conserve water, in the elderly, there is a decrease in total body water content associated with a reduction of plasma volume. These changes make the elderly much more sensitive to water overload or dehydration resulting in abnormal movement of solutes and, thereby, increasing the possibility of developing hypo- or hypernatremia.

However, an increase in vasopressin levels can also be found in aged people, where it induces water retention and hyponatremia and stimulates calcium release from bone, thus contributing to osteoporosis, as well as affecting the cardiovascular system and blood pressure, thus contributing to the development of hypertension.

For these diseases often associated with the elderly, vasopressin receptor antagonists represent a promising therapeutic tool. Interest in vasopressin has been renewed with the availability of vaptans, new, potent, orally active vasopressin receptor antagonists, initially developed for the treatment of various forms of hyponatremia (often related to vasopressin dysfunction) and proven to be safe in humans [109–111]. Evaluation of the specific aquaretic effect of vaptans in aged patients treated with this antagonist might have a profound impact in understanding the therapeutic effect of vaptan compounds.

Interestingly, vasopressin, the levels of which are increased after water deprivation, stimulates vascular superoxide production through activation of V1aR [112]. Accordingly, it has been shown that water deprivation increases ROS production in the somatosensory cortex, indicating that cerebrovascular dysfunction is related to oxidative stress [113].

### 5.1. Oxidative Signals and AQP2

Oxidative stress plays a key role in modulating renal functionality, including its diluting and concentrating ability during aging [114]. Oxidative stress increases the risk of developing several age-related diseases because ROS may alter cell signaling, leading to inflammation, apoptosis, and cellular senescence. During aging, significant increases in advanced glycosylation end products (AGE) and other oxidants have been reported in kidneys [115, 116]. Chronic inhibition of nitric oxidase synthase regulates renal water balance by reducing the expression of AQP2 [117, 118]. Importantly, oxidative stress is often associated with disorders linked to redox unbalance.

At a molecular level, ROS can oxidize selective amino acids on target proteins. Oxidative dependent modifications are being shown to be fundamental in transducing several intracellular signals controlling pleiotropic functions such as cell proliferation, apoptosis, autophagy, and membrane transport. These modifications result from reactions between ROS or reactive nitrogen species (RNS) and amino acid residues [26]. Oxidative modifications mainly occur through the switching of the sulfur in target cysteines. However, cysteines are not the only residues involved in oxidative modifications as methionine, lysine, arginine, threonine, and proline residues can also be oxidized to reactive carbonyls [119]. Oxidative sensitive modifications include carbonylation, nitrotyrosinylation, succinylation, S-sulfenation, S-nitrosylation, S-glutathionylation, and disulfide formation.

Reversible glutathionylation results from the reaction between glutathione and cysteine residues (PSSG) upon exposure to RS. S-Glutathionylation is recognized as a crucial modification by which cells translate local changes of reactive species [120].

Using a proteomic approach, Sandoval and coworkers revealed that vasopressin stimulation is associated with increased expression of different oxidative related proteins such as glutathione S-transferase [121]. This observation is likely to indicate that oxidative signaling may somehow play a role in controlling the physiological signal transduction cascade initiated by vasopressin. Studies from several groups have revealed, indeed, that antioxidant compounds such as *N*-acetylcysteine (NAC) rescued the reduction of AQP2 abundance observed in rats subjected to the bilateral ureteral obstruction (BUO) [122]. Conversely, treatment with the oxidant 4-hydroxy-2-hexenal (HHE) decreases the abundance of AQP2 and activates several kinases such as p38-MAPK and ERK [123], which have been proposed to phosphorylate AQP2 at S261 [124–126]. Phosphorylation at S261 is involved in AQP2 ubiquitylation and degradation [125, 127].

However, vasopressin is not the only factor regulating AQP2 expression. In this respect, it has been demonstrated that the oxidant HHE increases the expression of the transcription factors NF-*κ*B and the enzyme NOX4 [123], both involved in modulating the expression level of AQP2. Specifically, NF-*κ*B decreased *AQP2* mRNA and protein abundance [128]. Conversely, NOX4 promotes *AQP2* expression [129]. These findings strongly suggest that AQP2 abundance is the result of a balanced activity between NF-*κ*B and NOX4. However, how oxidative signals modulate the stability of the target proteins remains to be clarified. It appears conceivable that transient oxidative posttranslational modifications may mean there is a molecular signature translating oxidative information signaling and thus controlling the fate of target proteins. In this respect, some of the authors of this review have recently shown that AQP2 undergoes S-glutathionylation. It was also found that the increase in AQP2 glutathionylation is paralleled by higher ROS production. Conversely, low levels of ROS, measured in cells displaying low intracellular calcium concentration, secondary to the expression of the calcium-sensing receptor (CaSR), associates with reduced S-glutathionylation of AQP2 [130]. Whether or not S-glutathionylation of AQP2 is involved in the water imbalance observed during aging remains to be investigated.

## 6. Modulatory Actions of Food Antioxidant Phytocompounds on Aquaporins

A growing number of food phytochemicals are being found to exert antioxidant and anti-inflammatory actions. Thanks to their ability to interact with pivotal signaling pathways, a number of food and herbal phytochemicals have been found to impart health benefits modulating important cellular functions such as growth, differentiation, death, and volume homeostasis as well as redox, metabolic, and energy balance.

To date, a large number of biologically active phytochemicals have been identified, characterized, and eventually modified as natural sources of novel compounds to prevent, delay, or cure many human diseases. This is an important achievement since secondary prevention or adjunct therapy through dietary intervention is a cost-effective alternative for avoiding the large burden of health care, especially that associated with chronic illnesses.

Similarly to several other transport systems, AQPs are also modulated by a number of food bioactive phytocompounds [131, 132]. The modulatory effects exerted by beneficial dietary patterns, food phytochemicals, and herbal compounds on AQPs, in both health and disease, is a fast growing topic as their exploitation may help support current medical treatment options to improve the prognosis of several diseases. Flavonoid modulation of AQPs has been reported to ameliorate forms of cerebral and retinal edemas of different origins (AQP4) [132–135], lung injuries (AQP1) [136], and Sjögren syndrome-associated xerostomia (AQP5) [137], to inhibit ovarian tumor growth (AQP5) [89] and protect against UV-induced skin damage (AQP3) [63]. Curcumin influences choroid plexus AQP1 [138], ovarian AQP3 [139], and brain AQP4 and AQP9, reducing intracranial pressure in brain injury, inhibiting ovarian cancer cell migration, and reducing brain edema, respectively [140–142]. Modulation of AQP channel gating by curcumin has been recently reported in a paper describing the use of HeLa cells to investigate the effects of some antioxidant phytocompounds on AQP1, AQP3, AQP8, and AQP11 [60]. Resveratrol, a stilbene compound, was found to inhibit human keratinocytes and ameliorate the ischemia/reperfusion injury acting on AQP3 [64] and AQP4 [143], respectively. The chalcone compound phloretin also acts on the expression of AQP9 to exert its antioxidant and anti-inflammatory actions [144]. Genistein and daidzein, two isoflavonoids, were found to upregulate the expression of uterine AQP1 by increasing the responsiveness to estrogens [145]. The monoterpenoid carvacrol has been reported to reduce the intracerebral hemorrhage-induced brain edema by downregulating brain AQP4 [146]. Triterpenoids have been shown to act on the expression of AQP1 to reduce cancer cell migration, counteracting metastasis, as well as to ameliorate forms of allergic rhinitis and to downregulate kidney AQP2 to protect against renal failures [147]. Capsaicin was found to increase the expression level of submandibular salivary gland AQP5 to ameliorate salivary gland hypofunction [148, 149]. While useful information is already available, further important achievements are expected from the ongoing studies on the modulatory effects exerted by biologically active phytocompounds on the expression and function of AQPs.

## 7. Conclusions and Future Perspectives

Excess of ROS within the cells and reduction of NO bioavailability can largely promote cellular dysfunction, which is linked to the development of metabolic disorders, cardiovascular and renal diseases, frailty, and aging. Key roles for AQPs as peroxiporins in the signal transduction pathways underlying diverse cellular functions, such as differentiation, proliferation, or mobility, are suggested by the recent evidence of AQP-mediated H_2_O_2_ transport at the plasma or mitochondrial membrane level. Dysregulation of peroxiporin function can lead to oxidative stress and eventually cell death. Alterations in AQP-mediated ROS and/or NO transport are therefore assuming an increasing translational value in physiology and pathophysiology with promising nutraceutical and pharmacological implications. Indeed, modulation of the peroxiporin and/or NO channel function of AQPs at the vascular, hepatic, testicular, or renal level may prove to be valuable in preventing or treating cardiovascular (vascular stiffness/hypertension, atherosclerosis), metabolic, and reproductive (impaired sperm cell motility) diseases. Last but not least, further work is warranted to investigate the involvement of AQPs in the antioxidant and anti-inflammatory actions exerted by food phytochemical compounds in order to devise new strategies to promote health and improve aging.

## Figures and Tables

**Table 1:** Functional relevance of mammalian aquaporins in health and disease.

Physiological functions involving aquaporins
*Generation of fluids*
(i) Urine [150]
(ii) Cerebrospinal fluid [151]
(iii) Aqueous humor [152]
(iv) Sweat [21]
(v) Saliva [21]
(vi) Tears [21]
(vii) Bile [153]
(viii) Gastrointestinal juices [33]
(ix) Seminal fluid [154]
*Immune response and inflammation*
(i) Memory T-cell longevity [155]
(ii) Inflammatory response [156]
(iii) Dendritic cell maturation [157]
*Metabolic homeostasis and energy balance*
(i) Gluconeogenesis [158]
(ii) Triacylglycerol synthesis [158]
(iii) Ammonia detoxification via ureagenesis [159]
*Nervous system physiology*
(i) Multiple functions [151]
*Other functions*
(i) Apoptosis [160]
(ii) Oxidative stress [26]
(iii) Cell migration [161]
(vi) Cell volume homeostasis [162]
(v) Angiogenesis [163]

Pathological states involving aquaporins

(i) Cardiovascular diseases [164]
(ii) Renal concentration disorders [165]
(iii) Inflammatory diseases [156]
(iv) Cholestasis [153]
(v) Brain edema [151]
(vi) Cataract [162]
(vii) Immune system disorders (i.e., neuromyelitis optica) [166]
(viii) Malaria [167]
(ix) Obesity, diabetes, liver steatosis [158]
(x) Cancer [168]
(xi) Infertility [169]
